# Cholesterol activates BK channels by increasing KCNMB1 protein levels in the plasmalemma

**DOI:** 10.1016/j.jbc.2021.100381

**Published:** 2021-02-06

**Authors:** Anna N. Bukiya, M. Dennis Leo, Jonathan H. Jaggar, Alex M. Dopico

**Affiliations:** 1 Department of Pharmacology, Addiction Science, and Toxicology, College of Medicine, The University of Tennessee Health Science Center, Memphis, Tennessee, USA; 2 Department of Physiology, College of Medicine, The University of Tennessee Health Science Center, Memphis, Tennessee, USA

**Keywords:** MaxiK, slo1, vascular smooth muscle, BK beta1 subunit, BA, basilar artery, BFA, brefeldin A, BK, large conductance, CA, coronary artery, Ca^2+^-and voltage-gated potassium (channels), CLR, cholesterol, CRAC, cholesterol recognition amino acid consensus (motif), DM, dissociation medium, KO, knockout, MβCD, methyl-β-cyclodextrin, MCA, middle cerebral artery, NO^•^, nitric oxide, PBS, phosphate-buffered saline, wt, wild type

## Abstract

Calcium-/voltage-gated, large-conductance potassium channels (BKs) control critical physiological processes, including smooth muscle contraction. Numerous observations concur that elevated membrane cholesterol (CLR) inhibits the activity of homomeric BKs consisting of channel-forming alpha subunits. In mammalian smooth muscle, however, native BKs include accessory KCNMB1 (β_1_) subunits, which enable BK activation at physiological intracellular calcium. Here, we studied the effect of CLR enrichment on BK currents from rat cerebral artery myocytes. Using inside-out patches from middle cerebral artery (MCA) myocytes at [Ca^2+^]_free_=30 μM, we detected BK activation in response to *in vivo* and *in vitro* CLR enrichment of myocytes. While a significant increase in myocyte CLR was achieved within 5 min of CLR *in vitro* loading, this brief CLR enrichment of membrane patches decreased BK currents, indicating that BK activation by CLR requires a protracted cellular process. Indeed, blocking intracellular protein trafficking with brefeldin A (BFA) not only prevented BK activation but led to channel inhibition upon CLR enrichment. Surface protein biotinylation followed by Western blotting showed that BFA blocked the increase in plasmalemmal KCNMB1 levels achieved *via* CLR enrichment. Moreover, CLR enrichment of arteries with naturally high KCNMB1 levels, such as basilar and coronary arteries, failed to activate BK currents. Finally, CLR enrichment failed to activate BK channels in MCA myocytes from *KCNMB1*^*-/-*^ mouse while activation was detected in their wild-type (C57BL/6) counterparts. In conclusion, the switch in CLR regulation of BK from inhibition to activation is determined by a trafficking-dependent increase in membrane levels of KCNMB1 subunits.

Cholesterol (CLR) is an essential component of membranes in animal cells. The largest reservoir of cholesterol resides within the plasma membrane (1, 2). Here, CLR regulates the membrane’s physical state, microdomain formation, and the activity of membrane-spanning proteins, including ion channels (2, 3, 4, 5). Large-conductance, voltage- and Ca^2+^-gated K^+^ (BK) channels in the plasma membrane link membrane potential to cell Ca^2+^ homeostasis (6). Thus, BK channels are widely present in both excitable and nonexcitable cells controlling key physiological processes, such as endocrine secretion, neuronal firing, and smooth muscle contractility (6, 7, 8). Consistently, alterations in BK activity lead to multiple pathological conditions (9, 10).

The sensitivity of native BK currents to treatments that modify membrane CLR levels was first reported in cultured myocytes from human and rabbit aortas (11, 12) and since then in a wide variety of species and tissues, including mouse colonic epithelial cells (13), rat ureteric (14), uterine (15), and cerebrovascular myocytes (16), pituitary tumor GH3 cells (17), cardiomyocytes (18), bovine aortic endothelial cells (19), chicken hair cells (20), human myometrial myocytes in culture (21), melanoma IGR39 cells (22), and glioma cell lines (23). Studies on BK channel-forming alpha subunits reconstituted into artificial, two-three species phospholipid bilayers demonstrate that CLR presence in the bilayer depresses single-channel activity largely *via* reduction of channel open probability (16, 24, 25, 26). In *native* cell membranes, however, a given modification of membrane CLR levels has been reported to potentiate, inhibit, or even fail to affect BK currents (reviewed in 27).

Considering the critical role of elevated CLR in cardiovascular pathology, major efforts have been put forth for uncovering the molecular mechanisms that determine the mode of vascular smooth muscle BK current responses to increased CLR levels. In this tissue, BK channels consist of a homotetramer of channel-forming alpha (slo1) subunits that is accompanied by small accessory proteins of beta1 (KCNMB1) and gamma (LRRC26) types (6, 7, 28). The former increases the apparent calcium sensitivity of BK complexes and enables BK channel activation in response to a local increase in intracellular calcium near the BK channel’s calcium intracellular sensors (29, 30, 31). Upon activation, BK channels generate outward potassium currents that dampen the membrane potential toward its resting level and thus oppose depolarization-induced calcium entry and myocyte contraction (6, 29, 31).

In the present work, we employed a rat model of high-CLR diet, patch-clamp electrophysiology on freshly isolated cerebral artery myocytes, CLR *in vitro* enrichment, surface protein biotinylation followed by Western blotting, and KCNMB1 knockout (KO) mouse to demonstrate that the CLR effect on native smooth muscle BK current was determined by a CLR-driven increase in surface KCNMB1 protein. Our findings contribute to explain the differential outcomes from earlier studies on CLR modification of BK currents and open a new avenue for designing pharmacological interventions against unwanted effects of elevated CLR on vascular smooth muscle BK channel activity.

## Results

### *In vitro* and *in vivo* enrichment of MCA smooth muscle with CLR activates BK currents

First, we established the efficiency of the CLR enrichment *in vitro* procedure using fluorescence staining with filipin after a 20 min-long incubation of rat MCAs in MβCD:CLR complex. The cerebral artery smooth muscle layer within the tunica media was identified by a positive immunofluorescence staining against BK channel smooth muscle-specific KCNMB1 protein, which is scarce in the endothelium (Fig. S1, *A* and *B*) (32). In contrast, immunostaining against the endothelial cell marker platelet endothelial cell adhesion molecule-1 (CD-31) showed a fluorescence peak within the presumed tunica intima layer with scarce (if any) staining in tunica media. This fluorescence pattern is opposite to that of KCNMB1 labeling (Fig. S1, *A* and *C* and (33)). Moreover, nuclei and cytoplasm of BK KCNMB1-positive cells were in a perpendicular orientation, as opposed to CD-31-positive cells, which were oriented parallel to the artery side (Fig. S1*A* and (34)). The perpendicular orientation of cells relative to the artery side was used to identify smooth muscle cells in our filipin staining studies.

**Figure 1:**
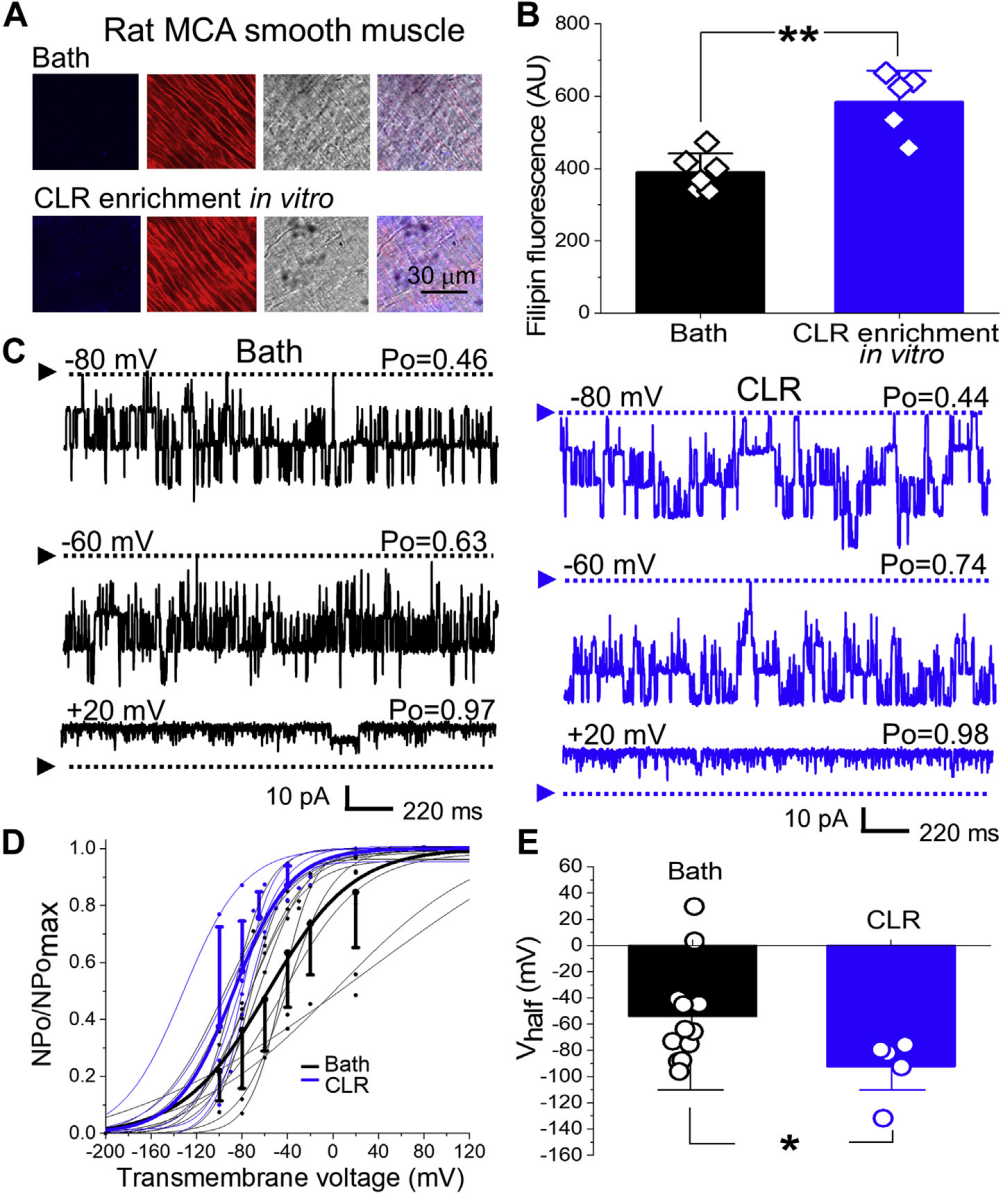
***In vitro* CLR enrichment increases smooth muscle CLR in rat MCA and activates BK channels.***A*, original snapshots of filipin-stained segments of rat MCAs following a 20 min-long CLR enrichment with 0.625 mM CLR. Within each row, panels reflect (from *left to right*): filipin staining (*blue*), plasmalemmal marker wheat germ agglutinin (*red*), smooth muscle layer image in visible light (*gray*), and an overlay of all three frames (*multicolor*). *B*, scattered data showing increase in filipin-associated fluorescence following a 20 min-long enrichment of rat MCA segments with 0.625 mM CLR. *p* = 0.0087 by two-tailed Mann–Whitney test. *C*, BK recordings from an I/O patch excised from rat MCA myocyte with naïve CLR content (*black*, number of channels N = 4) or CLR-enriched for 20 min prior to patch excision (*blue*, N = 4). Free [Ca^2+^] in the bath solution was set at 30 μM. Here and in all other figures, Po: open probability of a single channel. *D*, Boltzmann fitting of NPo/NPo_max_-V data from individual patches from rat MCA myocytes with naïve CLR (*black*) and following a 20-min-long enrichment with CLR prior to patch excision (*blue*). Averaged data for each group are depicted by thick *black* and *blue curves*, respectively. Here and in all other graphs, data are presented as mean ± SD. *E*, Scattered data showing decrease in V_half_ following *in vitro* CLR enrichment of rat MCA myocytes performed prior to patch excision. *p* = 0.0365 by two-tailed Mann–Whitney test.

Z-stack imaging of MCA smooth muscle layer detected a 1.5-fold averaged enhancement of filipin-associated fluorescence in the CLR-enriched arteries when compared with their counterparts with naïve CLR (*p* = 0.0087, Fig. 1, *A* and *B*). This finding was validated *via* biochemical detection of CLR level in MCA lysates (Fig. S2).

To determine whether CLR modifies the activity of native BK channels in rat MCA smooth muscle, we recorded BK currents in membrane patches from freshly isolated myocytes that were either loaded with CLR for ≥20 min prior to patch excision or incubated in CLR-free bath solution for the same time interval to maintain naïve CLR levels (control). Free Ca^2+^ in recording solutions was set at 30 μM to mimic calcium levels in the vicinity of the BK channel during myocyte contraction (30). Consistent with findings from other laboratories, BK channel open probability and V_half_ data exhibited a noticeable variability across patches (Fig. 1, *D* and *E*). A parallel shift of current–voltage curves along the voltage- (x-) axis has been reported for homomeric BK channels made of different isoforms (35, 36, 37, 38, 39), which may be explained by the complex structural bases underlying the gating behavior of this channel (40). We also observed variability in the steepness of the current–voltage curves across patches (Fig. 1, *D* and *E*). A reduced steepness of current–voltage curves is characteristic of the presence of accessory KCNMB1 subunits within the BK channel protein complex (41, 42). Thus, the variability in the current–voltage curve steepness is consistent with the very likely differential distribution of accessory KCNMB1 subunits within functional BK channels across patches. Indeed, the number of these accessory subunits can vary between 1 and 4 per functional BK channel, with BK current characteristics changing incrementally per each KCNMB subunit addition (reviewed in (43)). Consistent with previous reports (32, 44), V_half_ of BK current at 30 μM [Ca^2+^]_free_ averaged –53.9 ± 10.9 mV (Fig. 1, *C*–*E*). *In vitro* CLR enrichment of myocytes prior to patch excision robustly increased BK channel activity, as evidenced by a leftward shift in the NPo/NPo_max_-V curve along the x-axis with a consequent significant decrease in V_half_ (-92.2 ± 10.3 mV; *p* = 0.0365 when compared with naïve CLR) (Fig. 1, *C*–*E*).

**Figure 2:**
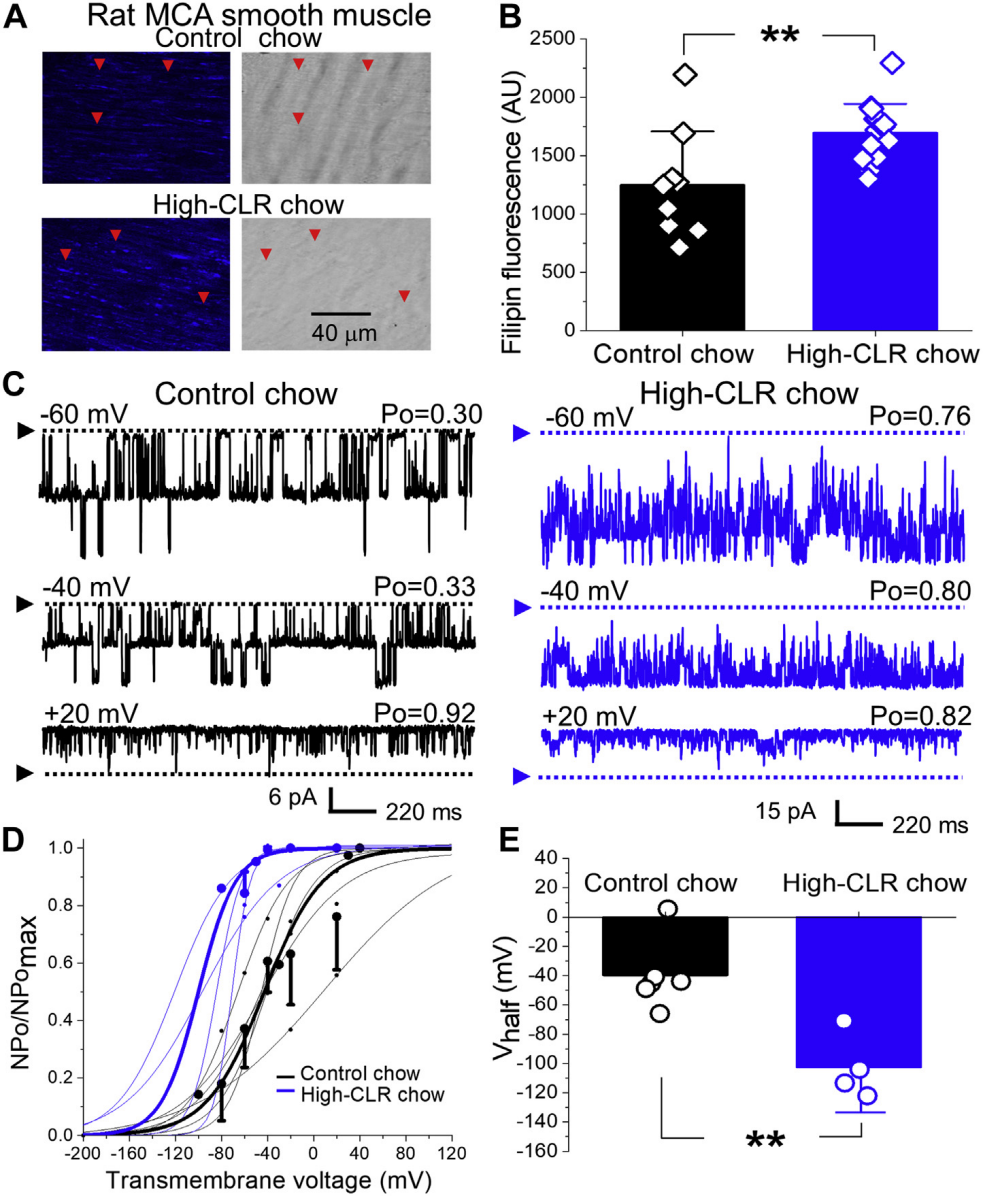
**High-CLR diet *in vivo* increases smooth muscle CLR in rat MCA and activates BK channels.***A*, original snapshots of filipin-stained segments of rat MCAs harvested after 29 weeks from placing 25 day-old rats on high-CLR diet (2% CLR). *Red arrows* point at the plasmalemma of individual myocytes. *B*, scattered data showing increase in filipin-associated fluorescence following enrichment of rat MCA segments with CLR in the course of high-CLR diet. *p* = 0.0027 by one-tailed Mann–Whitney test. *C*, BK recordings from an I/O patch excised from rat MCA myocyte from rats on control chow (*black*, number of channels N = 2) or on high-CLR chow offered for 29–32 weeks (*blue*, N = 6). Free [Ca^2+^] in the bath solution were set at 30 μM. *D*, Boltzmann fitting of NPo/NPo_max_-V data from individual patches from rat MCA myocytes in control chow-fed (*black*) and CLR chow-fed (*blue*) groups. Averaged data for each group are depicted by thick *black* and *blue curves*, respectively. *E*, scattered data showing decrease in V_half_ following *in vivo* accumulation of within rat MCA smooth muscle. *p* = 0.0095 by two-tailed Mann–Whitney test.

To ensure that the observed increase in BK channel activity was not exclusive to our particular method of CLR *in vitro* enrichment, we next conducted experiments in smooth muscle cell patches excised from MCAs that were harvested from rats on a high-CLR diet and from their counterparts on standard rodent chow (control). Subjecting rats to a high-CLR diet for 29–32 weeks significantly increased CLR levels in MCA smooth muscle layer (*p* = 0.0027, Fig. 2, *A* and *B*). Similar to CLR *in vitro* enrichment, this *in vivo* route of CLR delivery resulted in a statistically significant upregulation of BK currents with V_half_ decreasing from –39.8 ± 9.8 mV in control to –102.6 ± 11.2 in high-CLR chow (*p* = 0.0095, Fig. 2, *C*–*E*).

### CLR-driven increase in BK channel activity requires protein cellular trafficking

**Figure 3:**
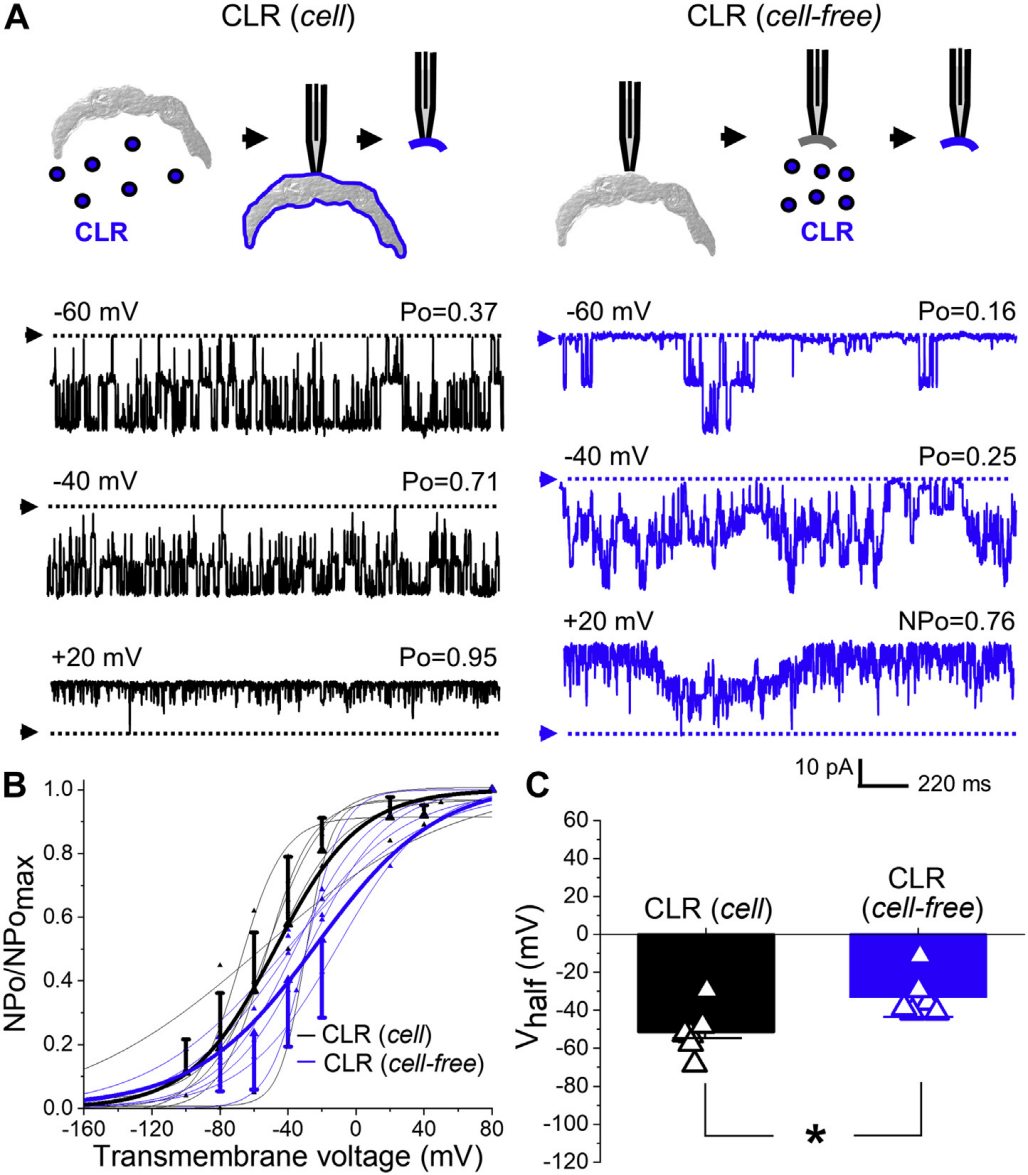
**Loss of cellular environment results in BK channel inhibition by CLR.***A*, BK recordings from an I/O patch from rat MCA myocyte excised following a 5 min-long enrichment of myocyte with CLR *in vitro* (*black*, number of channels N = 3) or excised and then enriched with CLR for 5 min (*blue*, N = 7). Free [Ca^2+^] in the bath solution was set at 30 μM. *B*, Boltzmann fitting of NPo/NPo_max_-V data from individual patches from rat MCA myocytes when patches are excised after (*black*) and before (*blue*) 5-min-long enrichment with CLR. Averaged data for each group are depicted by thick *black* and *blue* curves, respectively. *C*, scattered data showing increase in V_half_ following application of CLR-enriching media to excised patches as opposed to CLR enrichment of myocytes prior to patch excision. *p* = 0.0260 by two-tailed Mann–Whitney test.

Consistent with a recent report from our group (45), a time course of MCA *in vitro* enrichment with CLR showed a statistically significant increase in MCA CLR levels within 5 min of MCA incubation in CLR-enriching solution (Fig. S2). To establish a possible role of the intracellular environment and/or cell organelles other than the plasmalemma in the CLR-driven increase in BK channel activity, patch-clamp experiments were conducted on membrane patches that were perfused with CLR-enriching solution for 5 min *following* patch excision from the rat MCA myocytes. Remarkably, CLR enrichment of excised patches did not result in increased BK channel activity, a result that contrasts with that obtained after a 5 min-long enrichment of the intact myocyte with CLR immediately prior to patch excision (Fig. 3*A*). Moreover, cell-free enrichment of patches with CLR actually decreased BK channel activity, as evidenced by a rightward shift in NPo/NPo_max_-V curve along the x-axis and consequent significant increase in V_half_: 32.9 ± 4.6 mV versus -51.5 ± 5.2 mV in patches that were CLR-enriched prior to excision (*p* = 0.0260) (Fig. 3, *B* and *C*).

**Figure 4:**
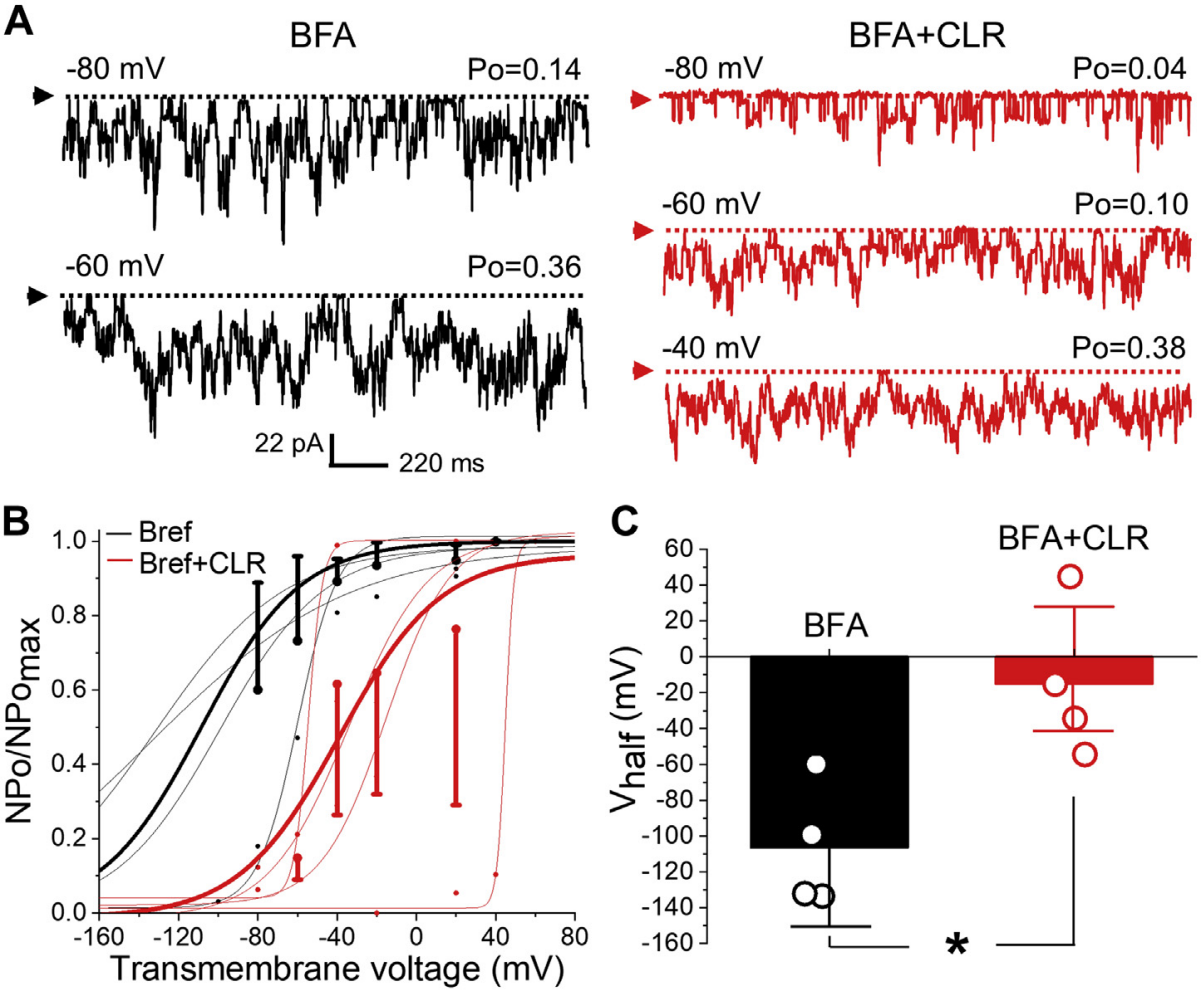
**Block of cellular trafficking switches BK channel activation by CLR enrichment to inhibition.***A*, BK recordings from an I/O patch from rat MCA myocyte excised following a 60 min-long incubation of myocyte in 10 μM brefeldin-A (BFA) without (*black*, number of channels N = 18) or with addition of CLR-enriching solution for 20 min at minute 40 of incubation (*red*, N = 17). Free [Ca^2+^] in the bath solution was set at 30 μM. *B*, Boltzmann fitting of NPo/NPo_max_-V data from individual patches from rat MCA myocytes subjected to BFA block of cellular trafficking without (*black*) and with subsequent addition of CLR-enriching solution (*red*). Averaged data for each group are depicted by thick *black* and *red curves*, respectively. *C*, scattered data showing increase in V_half_ when CLR enrichment of rat MCA myocytes was performed in the presence of BFA as opposed to BFA treatment alone prior to patch excision. *p* = 0.0286 by two-tailed Mann–Whitney test.

The fact that a 5 min *in vitro* incubation of myocytes in CLR-enriching media rendered effective increase in CLR levels of the myocyte yet it was not sufficient to evoke BK channel potentiation indicates that CLR diffusion across membranes cannot simply account for the CLR-driven activation of BK currents observed with longer incubation times. Moreover, the differential responses of BK channels to CLR enrichment of the intact cell versus the isolated membrane patch underscore that CLR-enrichment-evoked channel potentiation requires the intracellular environment and/or organelles additional to the plasmalemma. To test whether this regulatory role of the intracellular environment on CLR action involved actual protein trafficking between internal organelles, we utilized brefeldin A (BFA). BFA is a pan-inhibitor of protein transport from the endoplasmic reticulum to the Golgi complex (46), hence some nonspecific effects cannot be ruled out. In the presence of BFA, however, the fundamental phenotype of the BK channels under study was preserved (Fig. 4, *A* and *B*). Consistent with our hypothesis, block of endoplasmic reticulum–Golgi traffic with 10 μM brefeldin A (BFA; (47)) for at least 30 min prior to addition of CLR-enriching solution to isolated myocytes and patch excision failed to activate BK current but led to a decrease in BK channel activity: V_half_ reached –14.9 ± 21.4 mV when compared with –106.3 ± 17.3 mV observed in BFA-treated myocytes with naïve CLR level (*p* = 0.0286) (Fig. 4).

### CLR-driven increase in BK channel activity is enabled by KCNMB1 protein accumulation in the membrane

**Figure 5:**
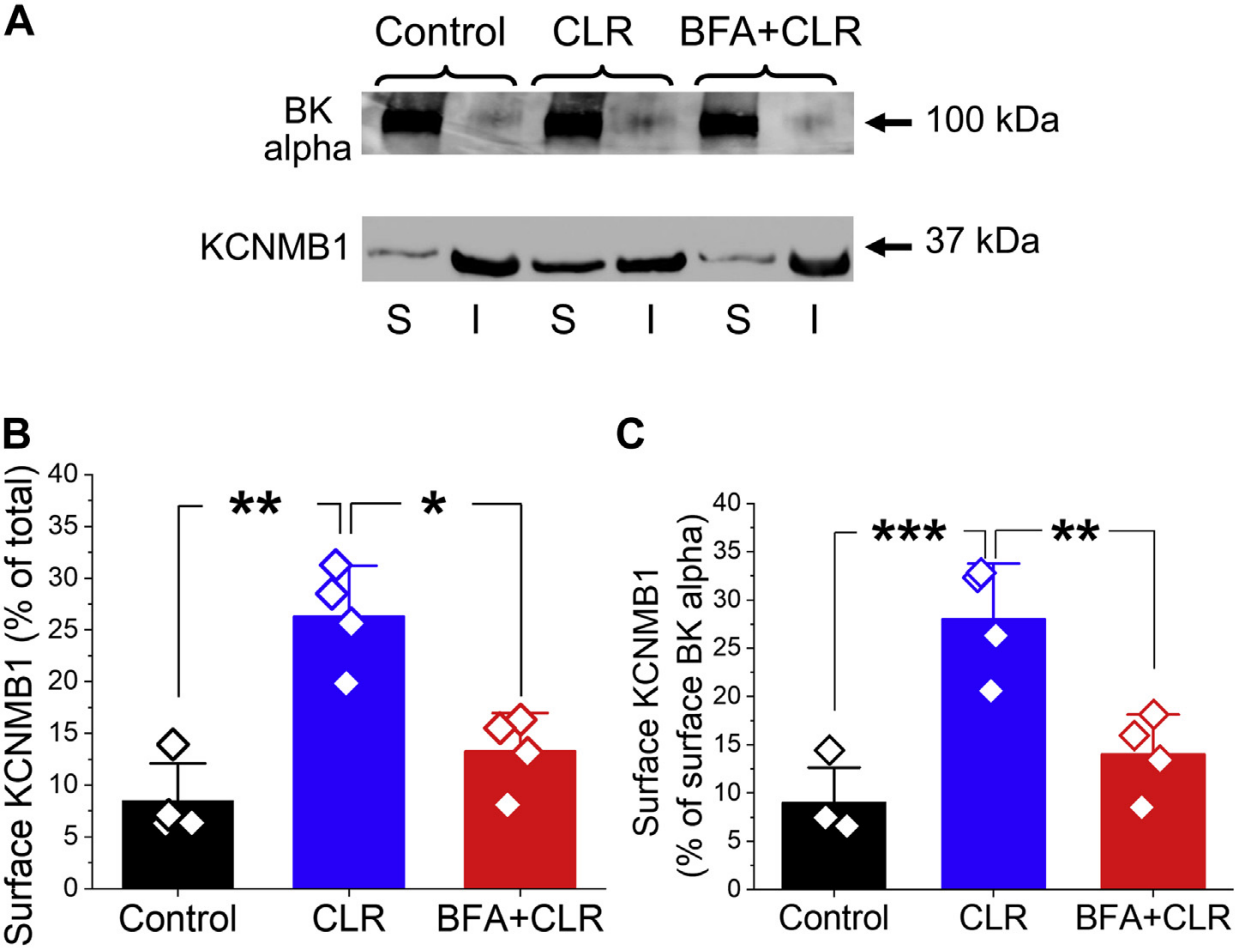
**CLR enrichment *in vitro* increases plasmalemmal abundance of KCNMB1 protein, but not BK channel alpha subunits.***A*, representative Western blot images showing that 20-min-long incubation of rat MCA segments in CLR-enriching solution results in upregulation of KCNMB1 cell surface (S) but not intracellular fraction (I). The CLR-driven upregulation of surface KCNMB1 is blunted by 10 μM BFA. In contrast, distribution of BK channel alpha subunits between S and I fractions remains unchanged by either treatment. *B*, scattered data showing surface KCNMB1 as percentage of total KCNMB1 protein level in control, after CLR enrichment, and after CLR enrichment in presence of 10 μM BFA. *p* = 0.0005 by one-way ANOVA with Tukey posttest; ∗∗∗*p* < 0.001, ∗∗*p* < 0.01. *C*, scattered data presented as a ratio of surface KCNMB1 over surface BK alpha subunit protein. *p* = 0.0007 by one-way ANOVA with Tukey post-test; ∗∗∗*p* < 0.001, ∗∗*p* < 0.01.

Smooth muscle-prevalent KCNMB1 accessory proteins in BK channel complexes increase the apparent calcium sensitivity and upregulate BK currents (6, 7), leading to the hypothesis that CLR-enrichment-induced potentiation of current is driven by enhanced modulation of BK channel-forming alpha subunits by KCNMB1 in the plasmalemma. Compared with BK alpha subunits, KCNMB1 is primarily an intracellular protein (47). In our hands, a 20 min-long incubation of rat MCAs in CLR-enriching solution significantly increased KCNMB1 plasmalemmal fraction when compared with MCAs with naïve CLR level (Fig. 5). Consistent with a KCNMB1-driven mechanism to explain the inability of CLR enrichment to increase BK channel activity in the presence of BFA (Fig. 4*C*), BFA prevented the CLR-driven increase in KCNMB1 plasmalemmal fraction (Figs. 5 and S3). In contrast, BK channel alpha subunit surface protein was similar in naïve CLR (93.5 ± 5.4%), upon CLR enrichment (94.3 ± 4.2%) and CLR enrichment in the presence of BFA (95.0 ± 3.5%) (*p* = 0.9008 by ANOVA for comparison of all three groups; *p* = 0.8857 by two-tailed Mann–Whitney test comparing naïve CLR versus CLR enrichment; *p* = 0.6857 by two-tailed Mann–Whitney test comparing naïve CLR versus CLR enrichment in the presence of BFA) (Fig. 5).

**Figure 6:**
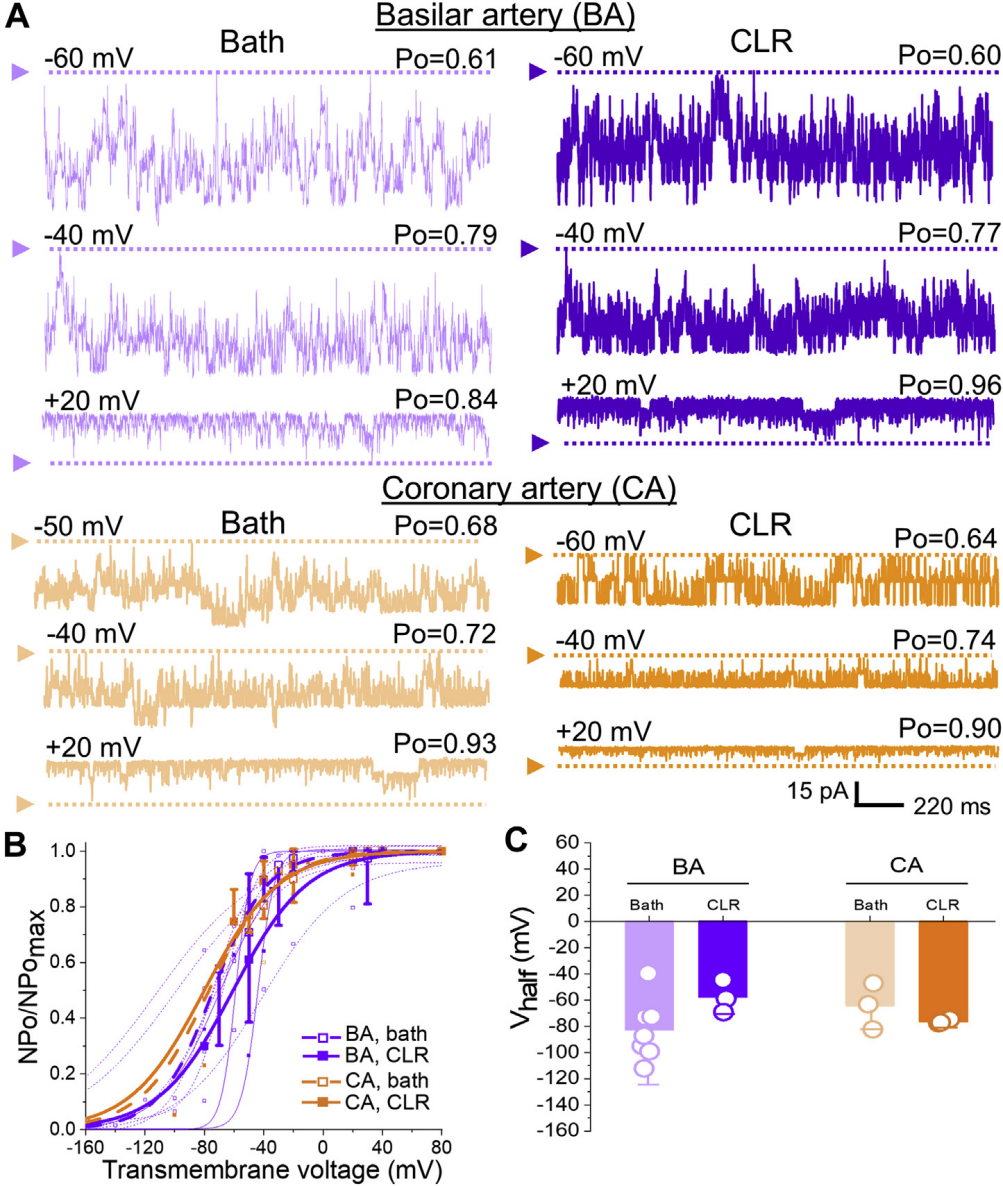
***In vitro* CLR enrichment of myocytes from basilar (BA) and coronary (CA) arteries that were reported to have the highest amounts of KCNMB1 protein in plasmalemma fails to activate BK currents.***A*, BK recordings from an I/O patch from rat BA (*top* records, *purple*) and CA (*bottom* records, *orange*) myocyte excised following a 20 min-long incubation of myocytes in bath (*left* recordings) of CLR-enriching solution (*right* recordings). Free [Ca^2+^] in the bath solution was set at 30 μM. BA bath record is from a patch with N = 8; BA CLR record is from a path with N = 7. CA bath record is from a patch with N = 6; CA CLR record is from a patch with N = 2. *B*, Boltzmann fitting of NPo/NPo_max_-V data from individual patches from rat BA and CA myocytes susbjected to either a 20 min-long incubation in bath or CLR-enriching solution prior to excision. Averaged data for each group are depicted by thick *purple* and *orange curves*, respectively. *C*, scattered data showing lack of decrease in V_half_ when CLR enrichment was performed on myocytes from BA or CA that are naturally high in KCNMB1 plasmalemmal fraction.

**Figure 7:**
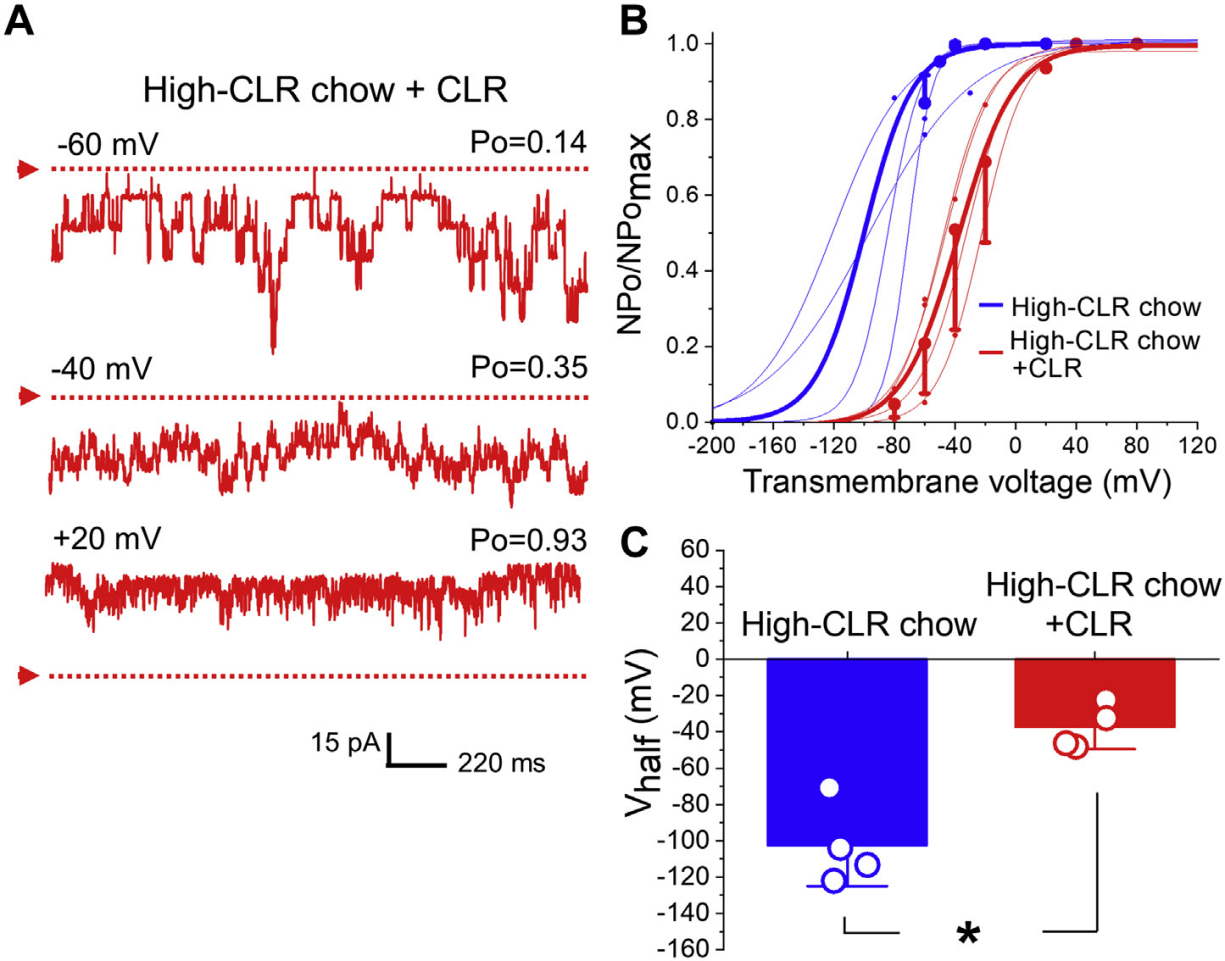
***In vitro* CLR enrichment of myocytes from MCA of rats on high-CLR diet inhibits BK channels.***A*, BK recordings from MCA of rat subjected to high-CLR diet for 29 weeks and to a 20 min-long CLR enrichment *in vitro* prior to patch excision. Free [Ca^2+^] in the bath solution was set at 30 μM, number of channels in the patch N = 13. *B*, Boltzmann fitting of NPo/NPo_max_-V data from individual myocyte membrane patches of rats on high-CLR chow without *in vitro* manipulation with CLR level (*blue*) and following a 20-min-long CLR enrichment *in vitro* prior to patch excision (*red*). Averaged data for each group are depicted by thick *blue* and *red curves*, respectively. *C*, scattered data showing increase in V_half_ when *in vitro* enrichment with CLR was performed on myocytes from rats receiving high-CLR chow when compared with rats on high-CLR chow without *in vitro* manipulations with CLR level. *p* = 0.0286 by two-tailed Mann–Whitney test.

To further test the hypothesis that increased KCNMB1 levels in the plasmalemma play a role in CLR-enrichment-driven potentiation of smooth muscle BK current, we evaluated the effect of CLR enrichment in myocytes isolated from basilar (BA) or coronary (CA) artery. These myocytes naturally have higher levels of plasmalemmal KCNMB1 when compared with their MCA counterparts (48). Consistent with the proposed major role for KCNMB1 levels in CLR potentiation of BK current, CLR enrichment failed to increase BK currents in BA and CA myocytes (Fig. 6) where KCNMB1 levels are high before CLR loading. Moreover, CLR enrichment of MCA myocytes from rats subjected to high-CLR diet and thus, having elevated KCNMB1 in plasmalemma (49) also failed to increase BK currents (Fig. 7). Indeed, CLR enrichment of these myocytes actually decreased BK channel activity, as evidenced by a rightward shift in NPo/NPo_max_-V curve along X-axis (Fig. 7*B*). Consistently, there was a significant increase in V_half_ that reached –37.4 ± 6.1 mV compared with –102.6 ± 11.2 mV observed in patches from myocytes of rats on high-CLR diet without subsequent *in vitro* enrichment with CLR (*p* = 0.0286) (Fig. 7*C*).

**Figure 8:**
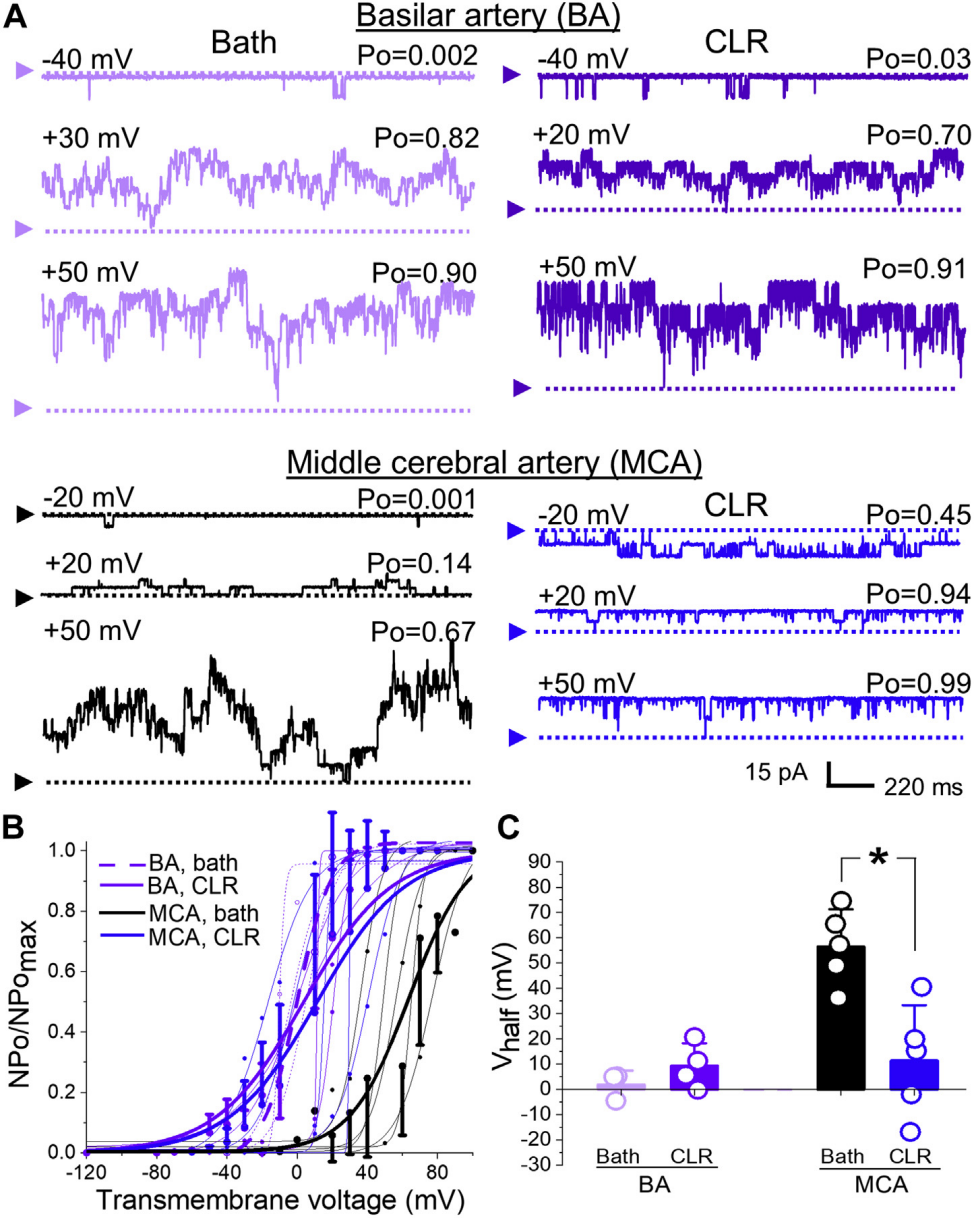
***In vitro* CLR enrichment of myocytes from BA and MCA reveals BK lack of effect and activation of BK currents, respectively, when recordings are performed at low calcium level.***A*, BK recordings from an I/O patch from rat BA (*top* records, *purple*) and MCA (*bottom* records, *black* and *blue*) myocyte excised following a 20 min-long incubation of myocytes in bath (*left* recordings) of CLR-enriching solution (*right* recordings). Free [Ca^2+^] in the bath solution was set at 3 μM. BA Bath record is from a patch with N = 8; BA CLR record is from a patch with N = 6. MCA bath record is from a patch with N = 8; MCA CLR record is from a patch with N = 6. *B*, Boltzmann fitting of NPo/NPo_max_-V data from individual patches from rat BA and MCA myocytes subjected to either 20-min-long incubation in bath or CLR-enriching solution prior to excision. Averaged data for each group are depicted by thick *purple* (BA) and *black* and *blue* (MCA) curves, respectively. *C*, scattered data showing lack of decrease in V_half_ when CLR enrichment was performed on myocytes from BA, while enrichment of MCA myocytes still rendered a decrease in V_half_. *p* = 0.0159 by two-tailed Mann–Whitney test.

To ensure that the mode of CLR-driven modifications in BK channel function was tuned by KCNMB1 plasmalemmal levels *per se* and not secondarily to an increase in BK channel basal activity, the effect of CLR enrichment *in vitro* was probed in patches that were enriched with CLR prior to excision into 3 μM [Ca^2+^]. This calcium level is substantially lower than that used in all other experiments (30 μM), yet still falls within the range of free [Ca^2+^] in the vicinity of cerebral artery myocyte BK channel during smooth muscle contraction (30). At 3 μM [Ca^2+^], BK channel V_half_ values in patches from both BA and MCA myocytes were lower than those at 30 μM [Ca^2+^] (Fig. 8*C* versus Figs. 1*E* and 6*C*). However, BA myocytes, which naturally have higher KCNMB1 plasmalemmal presence, exhibited lower V_half_ (1.9 ± 3.2 mV) when compared with V_half_ of BK currents from MCA myocytes (56.5 ± 6.6 mV, *p* = 0.0357). BA BK channels still failed to be activated by *in vitro* CLR enrichment of myocytes prior to patch excision while their counterparts from MCA myocytes (with lower KCNMB1 levels) were successfully activated by CLR enrichment (Fig. 8, *A* and *B*): MCA BK channel V_half_ decreased from 56.5 ± 6.6 mV in patches from myocytes with naive CLR to 11.4 ± 9.8 mV observed in patches from myocytes subjected to CLR enrichment prior to patch excision (*p* = 0.0159) (Fig. 8*C*).

**Figure 9:**
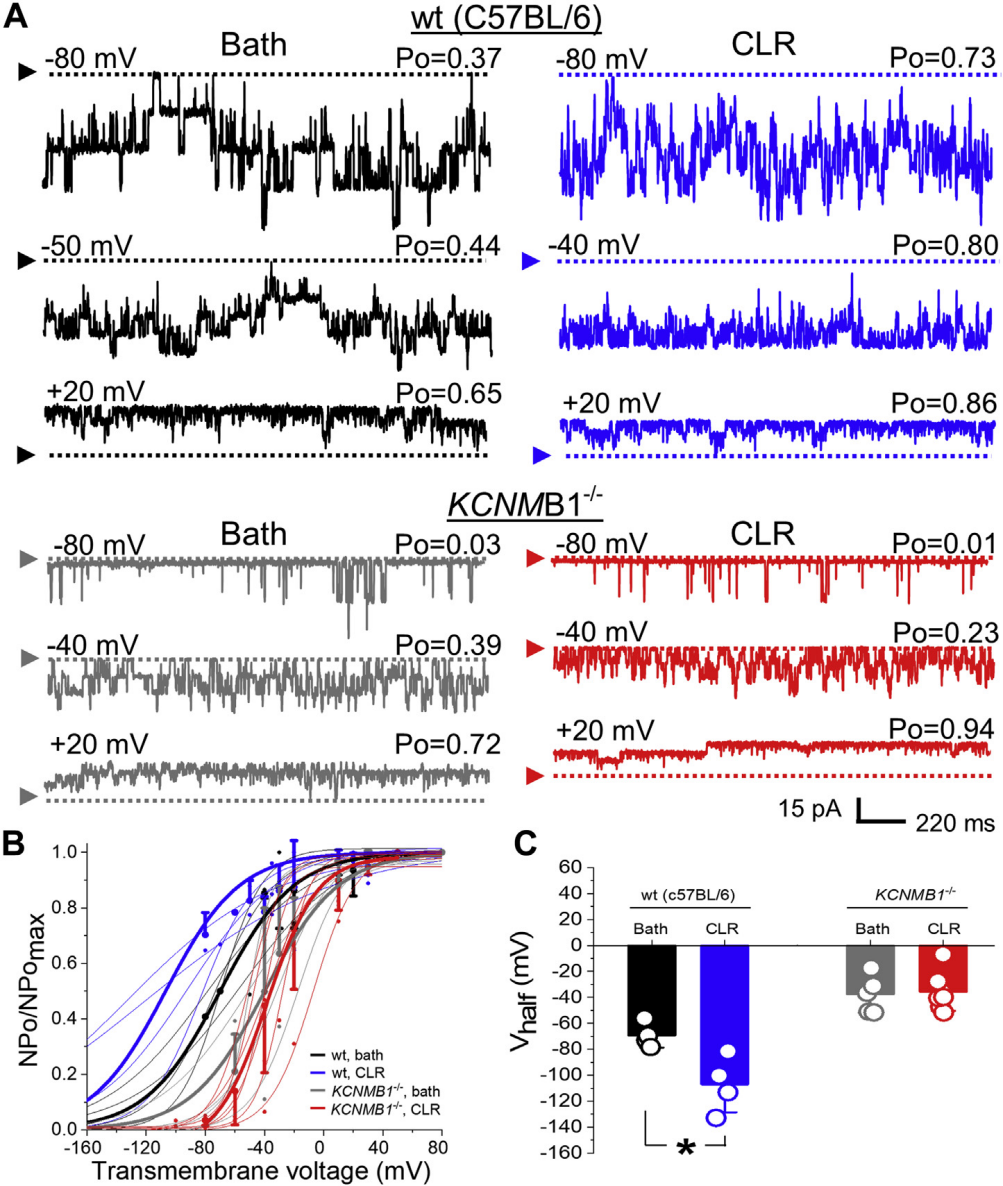
**CLR enrichment fails to activate BK channels in MCA myocyte patches from *KCNMB1* KO mice.***A*, BK recordings from an I/O patch from MCA of C57BL/6 (wild type, wt, *top traces*) and *KCNMB1*^-/-^ (KO, *bottom traces*) mouse myocytes. Traces in *black* and *gray* depict BK channel activity in patches with naïve CLR level, while traces in *blue* and *red* depict BK channel activity in patches from myocytes that were subjected to a 1 h-long enrichment with CLR *in vitro* prior to patch excision. Free [Ca^2+^] in the bath solution was set at 30 μM. Wt bath record is from a patch with N = 6; wt CLR record is from a patch with N = 5; *KCNMB1*^-/-^ bath record is from a patch with N = 4; *KCNMB1*^-/-^ CLR record is from a patch with N = 4. *B*, Boltzmann fitting of NPo/NPo_max_-V data from individual patches from MCA myocytes of wt and *KCNMB1*^-/-^ mouse with naïve CLR levels (*black* and *gray*) or from myocytes subjected to a 1 h-long incubation in CLR-enriching solution prior to patch excision (*blue* and *red*). Averaged data for each group are depicted by thick *black* (wt, naïve CLR), *gray* (*KCNMB1*^-/-^, naïve CLR), *blue* (wt, CLR enrichment), and *red* (*KCNMB1*^-/-^,CLR enrichment) curves. *C*, scattered data showing lack of decrease in V_half_ when CLR enrichment was performed on myocytes from *KCNMB1*^-/-^, while enrichment of MCA myocytes from wt mouse rendered a decrease in V_half_. *p* = 0.0286 by two-tailed Mann–Whitney test.

Finally, *in vitro* CLR enrichment of myocytes from wt mouse MCA resulted in BK channel activation, as evidenced by a leftward shift in NPo/NPo_max_-V curve along X-axis with a consequent significant decrease in V_half_: –106.9 ± 9.8 mV vs. -69.2 ± 4.7 mV in patches with naïve CLR levels (*p* = 0.0286) (Fig. 9). These data indicate that CLR-enrichment-driven potentiation of smooth muscle BK current is not limited to the rat species. Consistent with the critical role of KCNMB1 in driving CLR modification of BK channel function, however, the same *in vitro* CLR enrichment procedure failed to activate BK currents in MCA myocytes from *KCNMB1* KO mice (Fig. 9).

## Discussion

In the present study, we established for the first time the role of plasmalemma KCNMB1 levels in determining the response of channels to modulation by CLR. We show that in intact smooth muscle cells with low/moderate levels of KCNMB1 in plasma membrane, such as rat or mouse MCA myocytes, CLR enrichment upregulates KCNMB1 cell surface fraction and thus, increases BK channel activity. Moreover, this increase is independent of the CLR delivery method used (Figs. 1 and 2). However, when rat MCA myocyte membranes are either deprived of their intracellular environment and organelles (Fig. 3), or cellular trafficking of KCNMB1 is blocked (Fig. 4), or when surface KCNMB1 amount is already upregulated, *e.g.*, in case of BA and CA myocytes (Fig. 6), or by high-CLR diet on MCA (Fig. 7), CLR enrichment inhibits BK channels. Thus, CLR switch from BK current inhibition to activation is determined by CLR-driven increase in surface KCNMB1 levels.

Our findings solve a long-standing conundrum triggered by the differential outcomes of CLR enrichment on BK channel activity. Indeed, studies from reductionist systems (artificial lipid bilayers) unequivocally document that BK currents generated by homotetramers of alpha subunits from rat cerebral artery myocytes are inhibited by CLR (50, 51). This reduction of activity is not limited to alpha subunits of cerebrovascular myocyte origin but also reported with BK alpha subunits cloned from human brain (24). However, reduced BK channel activity in CLR-containing bilayers versus their CLR-free counterparts is also observed when KCNMB1 is present in the protein fraction subjected to reconstitution into bilayers (25). These findings are consistent with our present results indicating that the cellular environment and protein trafficking are necessary for CLR-induced activation of KCNMB1-containing BK channels (Figs. 3 and 4). In native myocyte membranes, exposure of freshly isolated cerebral artery myocytes to CLR-depleting treatment by using CLR-free methyl-beta-cyclodextrin results in increased open probability of BK channels in both C57BL/6 mouse (which possesses KCNMB1-containing BK channels) and *KCNMB1* knockout mouse (16). These reports seem to point at an inhibitory role of CLR on BK channel function when channels are located in a native environment and thus would appear to contradict our present findings. Two points should be made, however. First, as was already proposed for CLR modulation of BK channel’s sensitivity to alcohol, CLR effects on BK channel function might not be monotonic (52): first, the effect of depleting CLR levels that are naturally present in myocyte membranes may not mirror the effects of enriching native membranes with more CLR. Second, it is important to take into account the exact tissue type and artery type under examination, as even cerebral arteries from the same species exhibit differential levels of plasmalemmal KCNMB1 protein and therefore, differential sensitivity to modulators (48). In fact, our present data show upregulation of BK channel open probability by CLR enrichment of rat MCA, but not of BA or CA, myocytes (Fig. 1 versus 6). Preparations that contain differential sources of myocytes (such as mixtures of BA and MCA) could reasonably render various outcomes of CLR enrichment on BK function depending on the total number of data points obtained from a specific cerebral artery (BA versus MCA). Our present results are also in line with previous observations from another group that reported increased BK channel open probability in human smooth muscle from the atherosclerotic plaque when compared with arterial media segments (53). As atheromas are naturally enriched with CLR (54), they may mimic our conditions where there is upregulation of BK channel probability in rat MCA myocytes as a result of CLR accumulation *in vivo* during consumption of high-CLR chow (Fig. 2).

The central finding of our work is that a dynamic increase in the myocyte plasmalemmal fraction of KCNMB1 subunits switches the response of BK channel activity to CLR enrichment from inhibition to activation. Fluctuations in plasmalemmal levels of BK channel and accessory proteins have been shown to occur in the presence of pharmacological agents and/or physiological stimuli. In a mouse model, high-K^+^ alkaline diet increased the plasma membrane fraction of BK alpha subunits in distal nephrons (55). In primary cultures of rat hippocampal neurons, 6-h-long exposure to ethyl alcohol decreased the perimembrane (plasmalemmal and adjacent areas) fraction of BK channel alpha subunits with a detectable decrease in whole-cell BK current (56). In rat cerebral arteries, angiotensin promotes BK channel-forming alpha subunit internalization and thus a reduction in BK channel levels on the myocyte surface, resulting in vasoconstriction (57).

Unlike BK-channel-forming alpha subunits, KCNMB1 proteins have been shown to primarily reside inside the arterial myocytes with only a fraction of the total KCNMB1 pool residing in plasmalemma (46). Modulation of BK beta1 plasmalemmal level modifies cerebral artery contractility. In particular, rapid anterograde trafficking of KCNMB1 was shown to underlie depolarization- and nitric oxide (NO^•^)-induced activation of BK channels and resulting vasodilation (47, 58). Endothelin-1, a vasoconstrictor, inhibits KCNMB1 anterograde trafficking, thereby reducing BK channel activity and leading to vasoconstriction (59). While our earlier work detected an increase in plasmalemmal KCNMB1 amount in the course of high-CLR feeding of rats (49), current results document the consequences of such increases for BK channel function (Fig. 2). Data from our BFA studies seem to indicate that plasmalemma-intracellular traffic of KCNMB1 subunits with consequent increased levels of this subunit in the plasmalemma is required for CLR enrichment to increase smooth muscle BK current. Whether this CLR-driven increase in KCNMB1 plasmalemmal fraction is due to facilitated anterograde trafficking, diminished internalization/degradation of KCNMB1 protein, or both, remains to be formally tested. We favor the idea that facilitation of anterograde trafficking is involved: first, previous literature points at the importance of anterograde trafficking in NO^•^-driven increase KCNMB1 protein levels in cerebral artery myocyte plasmalemma (47, 58). Second, BFA is a most effective blocker of anterograde traffic (46). The fact that CLR enrichment of myocytes in the presence of BFA failed to activate BK channels (Fig. 4) points at the importance of protein traffic in this direction to CLR modification of BK current. If diminishing of retrograde traffic and KCNMB1 internalization were the primary mechanism of CLR-driven upregulation of BK channel activity, then BFA would not be so efficient in blunting CLR-driven activation of BK current and increase in plasmalemmal KCNMB1 fraction (Figs. 4 and 5).

The molecular mechanisms that enable CLR sensing by KCNMB1 remain elusive. Two nonmutually exclusive explanations are possible. First, KCNMB1 may directly bind CLR. Although CLR binding to KCNMB1 protein to KCNMB1 has not been reported, KCNMB1 amino acid sequence contains two cholesterol recognition amino acid consensus (CRAC) motifs. Although CRAC motifs are neither necessary nor sufficient to confer CLR-binding properties, they are considered as one of the major CLR-sequestering protein motifs (60). Thus, it is possible to speculate that CLR-bound KCNMB1 is preferentially trafficked to plasmalemma by the anterograde trafficking machinery. A second possibility is that CLR upregulates anterograde trafficking machinery independently of KCNMB1 interaction with CLR. Indeed, an increasing CLR gradient between endoplasmic reticulum and plasmalemma participates in protein sorting and directs anterograde trafficking of plasmalemma-bound proteins (61, 62). Thus, an increasing amount of plasmalemmal CLR may facilitate such sorting and traffic events. In our work, CLR effectively activated BK currents only when applied to the cell exterior, as opposed to the intracellular bilayer leaflet (*i.e.*, bath application to inside-out patches) (Fig. 3). This observation raises the question whether CLR has to be applied to the outer surface of the membrane for the effect to occur. Cholesterol is naturally present in both intracellular and extracellular leaflets of membrane bilayers (63). Moreover, the CLR molecule is highly hydrophobic; thus, CLR readily moves between membrane leaflets with an estimated flip-flop rate constant of 3 × 10^4^ s^−1^ (64, 65, 66, 67). This timescale is smaller than the BK channel gating constants. Thus, although the effector that enables CLR activatory action may reside in the extracellular membrane bilayer leaflet, experimental testing of this hypothesis remains to be conducted.

It can also be argued that the increase of KCNMB1 in plasmalemma is coincidental with CLR-driven improvement of functional coupling between alpha and KCNMB1 subunits that were already present within BK complexes at naïve CLR levels. This possibility cannot be excluded, yet it seems unlikely. First, CLR is not needed for the apparent increase in BK channel calcium sensitivity that is characteristic of KCNMB1 presence within the BK channel complex. Our earlier work on CLR-free planar lipid bilayers unequivocally demonstrated a leftward shift of NPo/NPo_max_-V curves along the x-axis when BK alpha subunit protein preparation was compared with a preparation combined with KCNMB1 (supplemental material in (25)). Second, if CLR enrichment were to improve intersubunit coupling within an existing BK complex, CLR enrichment of excised membrane patches would have rendered BK current activation, which is contrary to our results (Fig. 3). Whether the KCNMB1 pool recruited by elevated CLR is structurally and functionally similar to the KCNMB1 pool present in plasmalemmal at naïve CLR levels remains unknown.

Another novel observation in our study was that widely reported inhibition of homomeric (only consisting of alpha subunits) BK channels by CLR (27) was totally overridden by the activatory effect of KCNMB1 elevation in plasmalemma. CLR-induced inhibition of homomeric BK channels is enabled by multiple CRAC motifs within the BK alpha subunit cytosolic tail domain (51). Our present findings suggest that CLR-sensing CRACs in the BK alpha subunit are prevented from sensing CLR when high amounts of KCNMB1 protein are present and/or the degree of CLR-driven activation of BK currents by KCNMB1 recruitment exceeds the degree of inhibition *via* alpha subunit CRACs.

In our present study on native BK channels in their native environment (*i.e.*, the vascular myocyte membrane), we observed a CLR-driven decrease in V_half_ (Figs. 1 and 2, and wt mouse data in Fig. 9), an increase in V_hal_f (Figs. 3, 4 and 7), and no change in V_half_ (Fig. 6, and *KCNMB1*^*-/-*^ data in Fig. 9). While a switch from CLR-induced activation to inhibition of BK currents can be explained by upregulation of KCNMB1 plasmalemmal fraction and unmasking of an inhibitory effect in the absence of KCNMB1 upregulation, the lack of an expected inhibitory effect of CLR enrichment requires further investigation. Several observations, however, merit discussion. First, lack of change in V_half_ of BK currents was either observed in rat vascular tissue that was naturally enriched with KCNMB1 (such as BA and CA in Fig. 6) or in a genetically altered mouse model (*KCNMB1*^*-/-*^ in Fig. 9). Thus, there may be vascular territory and species specificity in BK alpha subunit isoforms, with this specificity resulting in a diminished response to CLR elevation. Second, and most important, even in the absence of a statistically significant change in V_half_ in the aforementioned experimental specimens, CLR enrichment always resulted in apparent shallowing of the slope in Boltzmann fits of NPo/NPo_max_-V curves. This shallowing merits quantification and further investigation, as it may lead to discovery of gating mechanisms that underlie CLR sensing by BK channel subunits. However, this finding is consistent with the idea that CLR may act as an isolator and thus somewhat diminish voltage sensing by BK channels (68).

In summary, our study has unveiled the critical role of KCNMB1 plasmalemma fraction in tuning BK current responses to CLR elevation. Involvement of accessory subunit trafficking into defining the mode of BK current modification by CLR enrichment explains the seemingly controversial previous literature on this topic and adds another layer of complexity to our understanding of CLR effects on ion channel function at the cellular level.

## Experimental procedures

### Ethical aspects of research

The care of animals and experimental protocols were reviewed and approved by the Animal Care and Use Committee of the University of Tennessee Health Science Center, which is an institution accredited by the Association for Assessment and Accreditation of Laboratory Animal Care International.

### CLR enrichment of the biological specimens

For CLR enrichment, we followed a previously described methodology (45). Methyl-beta-cyclodextrin (MβCD) at 5:0.625 mM steroid complex ratio (8:1 M ratio) was prepared in phosphate buffered saline (PBS) and an electrophysiology bath solution for arteries and myocytes/membrane patches, respectively. To ensure MβCD saturation with CLR, the solution was shaken at 37 °C overnight and filtered prior to its application to specimens (51).

### Fluorescence staining of rat middle cerebral artery (MCA) wall

Rats were decapitated under deep anesthesia with isoflurane. Arteries were dissected out under a microscope. Arteries were fixed in 4% paraformaldehyde for 30 min in the dark at room temperature. Permeabilization was performed using 0.5% Triton-100 in PBS for 10 min. For images validating the *in vitro* enrichment with CLR, arteries were stained with Alexa Fluor 594-conjugated marker of plasmalemma wheat germ agglutinin (Invitrogen) at a 1:200 dilution in the dark (room temperature, 30 min). Following washout, arteries were stained with a 25 μg/ml filipin solution for 1 h in the dark. Filipin solution was prepared by diluting 10 mg/ml filipin stock in dimethyl sulfoxide, which was stored at −20 °C for no longer than 1 week. After washing out the filipin-containing solution, arteries were briefly rinsed with deionized water and immediately mounted between a cover slip and slide glass using ProLong Gold antifade reagent (Invitrogen). Mounted specimens were dried at room temperature in the dark for 24 h and then sealed along coverslip edges with clear nail polish. Sealed slides were stored at −20 °C in the dark.

### Fluorescence data acquisition

Specimens were imaged using an Olympus FV-1000 laser scanning confocal system (Olympus). Filipin and wheat germ agglutinin fluorescence images were obtained using the 405 and 561 nm laser lines, respectively. Imaging was performed using a z-stack function, with the thickness of the step set at 1 μm. Stacks also contained recordings of the images obtained within the visible light spectrum. Within each artery, three areas were randomly chosen for imaging.

### Rat feeding with high-CLR chow

Twenty-five day-old (≈50 g of weight) male Sprague-Dawley rats were offered a high-CLR diet (2% cholesterol) in standard rodent food (Harland-Teklad) *ad libitum*. An equivalent (age, weight) group of male rats was fed control chow (*i.e.*, isocaloric to high-CLR) from the same supplier. Rats were used for experimentation after 29–32 weeks on control or high-CLR chow.

### Myocyte isolation

For myocyte isolation, male mice (8–12 week-old) and rats (250 g or at the end of high-CLR chow feeding) were used. On the day of the experiment, animals were decapitated under deep anesthesia with isoflurane. Arteries of interest were dissected out, cut into small pieces, and placed into an ice-cold dissociation medium (DM) with the following composition (mM): 0.16 CaCl_2_, 0.49 EDTA, 10 HEPES, 5 KCl, 0.5 KH_2_PO_4_, 2 MgCl_2_, 110 NaCl, 0.5 NaH_2_PO_4_, 10 NaHCO_3_, 0.02 phenol red, 10 taurine, 10 glucose. Next, arteries were transferred into polypropylene tubes with 3 ml DM containing 0.03% papain, 0.05% bovine serum albumin, and 0.004% dithiothreitol. Tubes were incubated at 37ºC for 10 min in an agitating water bath at 30 oscillations/min. Then, the supernatant was discarded, and the tissue transferred to a polypropylene tube with 3 ml DM containing 0.06% soybean trypsin inhibitor, 0.05% bovine serum albumin, and 2% collagenase (26.6 units/ml). The tube was incubated again in a shaking water bath at 37ºC and 30 oscillations/min for 10 min. Mouse arterial rings were initially put in 3 ml DM containing 0.00075% papain, 0.05% bovine serum albumin, and 0.004% dithiothreitol at 37ºC for 7 min in a shaking water bath at 30 oscillations/min. The tissue was then transferred to 3 ml DM containing 0.06% soybean trypsin inhibitor, 0.05% bovine serum albumin, and 2% collagenase (26.6 units/ml), and incubated under similar conditions for 7 min. Finally, either the rat or mouse artery tissue pellet was transferred into a tube with 3 ml of DM containing 0.06% soybean trypsin inhibitor. Tissue-containing DM was pipetted using a series of borosilicate Pasteur pipettes having fire-polished, diminishing internal diameter tips. The procedure rendered a cell suspension containing relaxed, individual myocytes (≥5 myocytes/field using a 20X objective) that could be identified under an Olympus IX-70 microscope (Olympus America). The cell suspension was stored in ice-cold DM containing 0.06% bovine serum albumin, and the cells were used for patch-clamp recordings up to 3 h after isolation.

### Electrophysiology experiments on freshly isolated rat/mouse arterial myocytes

BK currents at single-channel resolution were recorded from excised, inside-out (I/O) membrane patches. Both bath and electrode solutions contained (mM) 130 KCl, 5 EGTA, 1.6 HEDTA, 10 HEPES, 5.59 CaCl_2_ ([Ca^2+^]_free_≈30 μM), 2 MgCl_2_, pH 7.35. For experiments with [Ca^2+^]_free_≈3 μM, both bath and electrode solutions contained (mM) 130 KCl, 5 EGTA, 1.6 HEDTA, 10 HEPES, 4.49 CaCl_2_, 2.44 MgCl_2_, pH 7.35. Nominal free Ca^2+^ was calculated with MaxChelator Sliders. Patch-recording glass electrodes were fire-polished on a microforge WPI MF-200 (World Precision Instruments) to give resistances of 5–9 MΩ when filled with electrode solution. An agar bridge with Cl^-^ as the main anion was used as ground electrode. When required by experimental design, excised patches were exposed to a stream of CLR-containing or CLR-free bath solution that was applied onto the patches by using a computerized and pressurized OctaFlow system (ALA Scientific Instruments) *via* a micropipette tip with an internal diameter of 100 μm. Ionic current was recorded with an EPC8 amplifier (HEKA) at 1 kHz using a low-pass, eight-pole Bessel filter (model 902LPF; Frequency Devices). Data were digitized at 5 kHz using Digidata 1320A and pCLAMP 8.0 (Molecular Devices). For a proper comparison with data previously obtained by us and others, all studies were conducted at room temperature (20–22º C).

### Surface protein biotinylation and western blotting

Following artery dissection and 20-min-long enrichment with CLR *in vitro*, plasmalemmal protein biotinylation was performed at 4 °C using the Pierce Cell Surface Protein Isolation kit (Thermo Fisher Scientific) following the manufacturer’s instructions and as published earlier (47). The purified surface protein fraction was analyzed by Western blotting as follows. Purified surface protein fraction for biotinylation (50 μg/lane) was separated on a 4–15% SDS–polyacrylamide gel and transferred onto polyvinylidene difluoride (PVDF) membranes. Membranes were then blocked with 5% nonfat dry milk made in Tris-buffered saline (TBS) containing 0.1% Tween 20 for 2 h. Then, membranes were incubated with appropriate primary antibodies overnight at 4 °C in TBS with 0.1% Tween 20 (TBS-T) and 5% nonfat dry milk. Membranes were subsequently incubated with appropriate horseradish peroxidase–conjugated secondary antibodies (1:10,000 dilution; EMD Millipore) for 1–2 h at room temperature. Proteins were visualized using the SuperSignal West Pico Chemiluminescent Substrate kit (Thermo Fisher Scientific). A rabbit polyclonal anti-KCNMB1 antibody (1:500 dilution; ab57219; Abcam) and mouse monoclonal anti-slo1 antibody (1:1500 dilution; University of California, Davis/National Institutes of Health NeuroMab) were used to detect KCNMB1 and slo1 proteins, respectively (47). Blots were cut at ∼50 kDa to allow simultaneous probing for both BK α and β1 subunits. Proteins were visualized using SuperSignal West Pico Chemiluminescent Substrate (Thermo Fisher Scientific). Band intensities were analyzed using Quantity One software (Bio-Rad).

### Chemicals

Cholesterol was purchased from Avanti Polar Lipids. All other chemicals were purchased from purchased from Sigma-Aldrich.

### Data analysis

Patch-clamp and fluorescence data analyses were performed by an experimenter who was blind to the specimen’s control or experimental group source.

Fluorescence was quantified using built-in function in FV10-ASW 3.1 software (Olympus American Inc) as described (34). Within each z-stack, the sharpest image in visible light spectrum (filipin-stained specimens) or the sharpest DAPI signal (anti-KCNMB1- and anti-CD31-stained specimens) pointed at the focal plane used for fluorescence analysis. The filipin fluorescence corresponding to the plasmalemma–subplasmalemma region was obtained following direct superposition of the myocyte fluorescent image with the corresponding image of the myocyte under visible light. Within each imaged area, the fluorescence from three myocytes was measured and averaged to represent a single data point.

Electrophysiological data were analyzed with built-in function in Clampfit10.6 (Axon). NPo was used as index of channel steady-state activity, where NPo=N (number of channels in the patch) x Po (single channel open probability). NPo was obtained from ≥30 sec-long recordings at each voltage/experimental condition. The number of channels in the patch was determined after applying a depolarizing voltage step to +20 or up to +80 mV, depending on free [Ca^2+^] level at the intracellular patch leaflet. At these voltages, and in the respective presence of 30 or 10 μM free [Ca^2+^], smooth muscle BK channels reach almost maximal activation, with Po approaching 1 (69). Thus, N can be clearly determined from the number of opening levels. NPo/NPo_max_ data were fitted to a Boltzmann function to obtain V_half_, that is, the voltage needed to obtain half-maximal activity. In all fittings, channel activity was assumed to be absent at –300 and -250 mV while being at its maximal level at 200 and 300 mV.

Western blot data were analyzed using a free download of ImageJ software.

Further analysis, plotting, and fitting of data were conducted using Origin 2020 (Originlab Corp) and InStat 3.0 (GraphPad Software Inc). Differences between the groups were considered statistically significant when *p* < 0.05, details of statistical analysis are presented in figure captions. Data are expressed as mean±SD. Unless specified otherwise, each data point represents a single patch/Western blot membrane. Each patch was obtained from a separate myocyte and no more than three patches were obtained from one animal donor. Data points from the same animal donor were included into separate control/experimental groups so that individual data points within each group represent biological replicates.

### Data availability

Data are available upon request (abukiya@uthsc.edu).

## Supporting information

This article contains supporting information (16).

## Conflict of interest

The authors declare that they have no conflicts of interest with the contents of this article.
