# Supplementary material for: Cholesterol activates BK channels by increasing KCNMB1 protein levels in the plasmalemma
Source: J Biol Chem. 2021 Feb 6;296:100381. doi: 10.1016/j.jbc.2021.100381 (PMC7950327; doi:10.1016/j.jbc.2021.100381)
Supplement: Supplementary material [file mmc1.pdf]

SUPPORTING MATERIALS AND METHODS

Immunofluorescence labeling and confocal imaging. Staining procedures were performed following standard approaches as follows. To account for possible inter-experimental variability in fluorescence signal intensity, staining during each experiment was performed on specimens in parallel. Middle cerebral arteries (MCAs) of rats were dissected out, rinsed with phosphate buffered saline and fixed with 4% paraformaldehyde on ice for 30 min. MCAs were permeabilized with 0.5% Triton X-100 in phosphate buffered solution (PBS) at room temperature for 30 min. After thoroughly washing, blocking was carried out in an antibody dilution buffer supplemented with 50 µL/mL goat serum on ice for 30 min. The antibody dilution buffer consisted of 1% bovine serum albumin in PBS. Arteries were incubated at room temperature for 2 hours in the following primary antibodies: rabbit polyclonal antibody against the KCNMB1 protein (5:1,000; PA1-924, Thermo Fisher Scientific) or anti CD-31 (5:1,000, ab33858, Abcam). After primary antibody washout, the arteries were incubated at room temperature in the dark for 2 hours in a goat anti-rabbit preabsorbed secondary antibody conjugated with Cy5 (1:1,000, ab6564, Abcam) and goat anti-mouse preabsorbed secondary antibody conjugated with Alexa488 dye (1:1,000, ab6879, Abcam). After washout, cellular nuclei were stained with DAPI (Life Technologies Corporation) following manufacturer's instructions. After each stage, washing with PBS was performed at room temperature 3 times for 5 min using a 96-well plate and a benchtop orbital shaker (VWR Scientific Products). The cover slips were mounted using the ProLong AntiFade kit (P10144, Invitrogen). The cover slips were then dried for 24 hours at room temperature in the dark, and subsequently sealed using clear nail polish. The specimens were imaged using 40x, imaging was performed using the 405 nm (DAPI), 488 nm (Alexa488), and 635 nm (Cy5) laser lines of the Olympus FV-1000 laser scanning confocal system. Sequential line acquisition was used to minimize the probability of fluorescence emission crossover. The acquisition settings of the confocal microscope system remained unchanged throughout the imaging of all immunostained specimens. Z-stacks were obtained for each artery segment, with the step of 1 μm. The fluorescence was quantified using a built-in function in FV10-ASW 3.1 software (Olympus American Inc.). Three artery segments of equal size were imaged from each artery. For fluorescence quantification, one layer of an artery segment within a z-stack was selected based on the sharpness of the DAPI nuclear staining. Background fluorescence outside the artery edge was subtracted from the mean pixel intensity of each artery segment. Resulting values were used for statistical analysis using InStat 3 software (GraphPad).

Determination of cholesterol (CLR) level in the artery tissue. For the time course of CLR enrichment, arteries were dissected out and subjected to incubation with 5 mM methyl-β-cyclodextrin (MβCD) : 0.625 mM CLR complex in PBS for various time intervals (5, 20, 30, and 60 minutes). In parallel, time-matched control MCAs were incubated in CLR-free PBS. Artery tissue was homogenized as previously described (16). Protein and CLR levels were determined using the Pierce BCA Protein Assay Kit (Thermo Scientific) and Amplex Red Cholesterol Assay Kit (Molecular Probes), respectively. Kits were used following manufacturer instructions. Protein and CLR readings were performed with a microplate reader (Synergy) using absorbance (562 nm) and fluorescence (excitation/emission = 540/590 nm) reading functions, respectively.

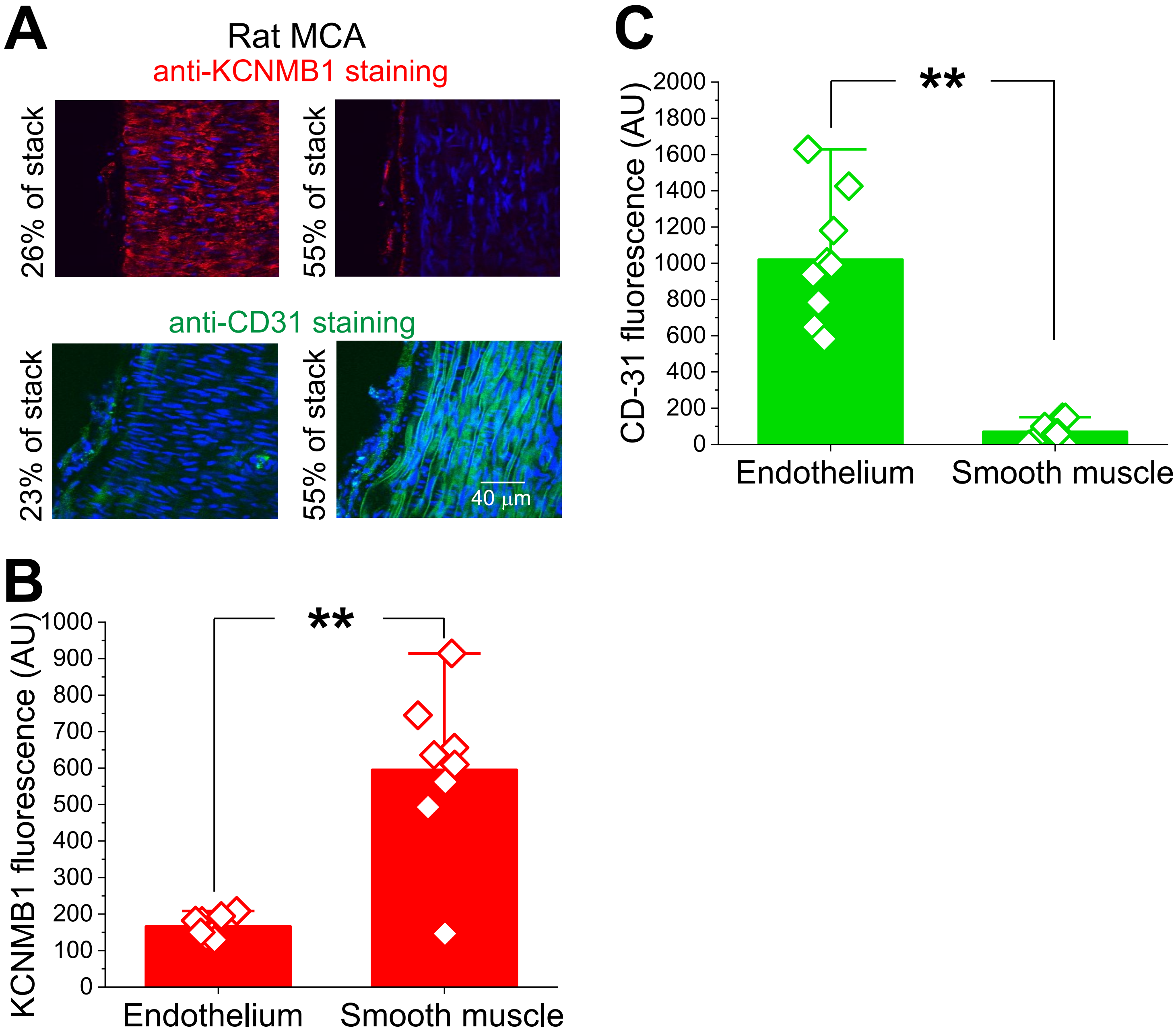

Supporting Figure 1. Identification of cerebral artery smooth muscle cells. A. Original snapshots showing preferential location of KCNMB1- (top row) as opposed to CD-31-associated fluorescence signals in different layers of a rat middle cerebral artery (MCA) z-stack. B. Scattered graph shows fluorescence intensity of different layers within rat MCA following immunostaining with anti-KCNMB1-specific antibody. Here and in (C), AU: arbitrary units; data are from 3 separate arteries that were stained on 2 independent experimental occasions. P=0.003 by two-tail Mann-Whitney test. C. Fluorescence intensity of different layers within rat MCA following immunostaining with anti-CD-31-specific antibody. P=0.001 by two-tail Mann-Whitney test.

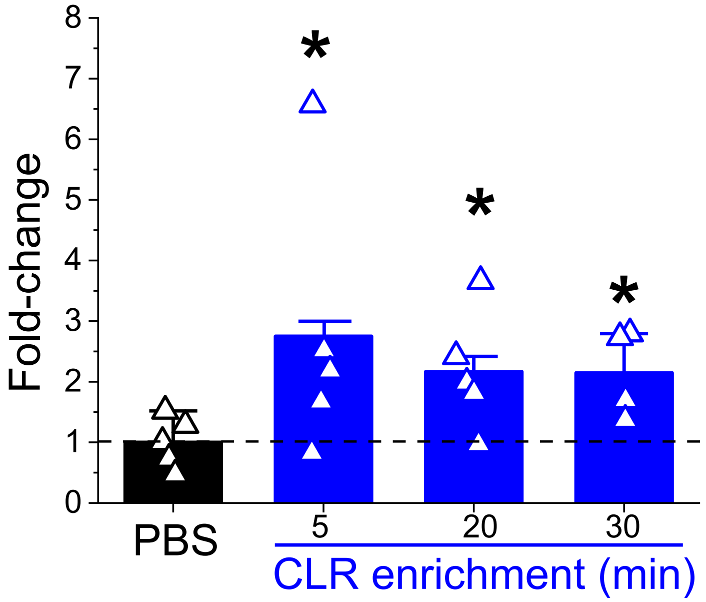

Supporting Figure 2. Time-course of *in vitro cholesterol (*CLR)-enrichment of rat cerebral arteries. Horizontal dashed line depicts averaged level of naïve CLR. For 5 min of in vitro enrichment with CLR, p=0.0278; for 20 min p=0.0411; for 60 min, p=0.0159 by one-tail Mann-Whitney test when compared to fluctuations of naïve CLR levels in cerebral arteries from different rats over averaged naïve CLR value.

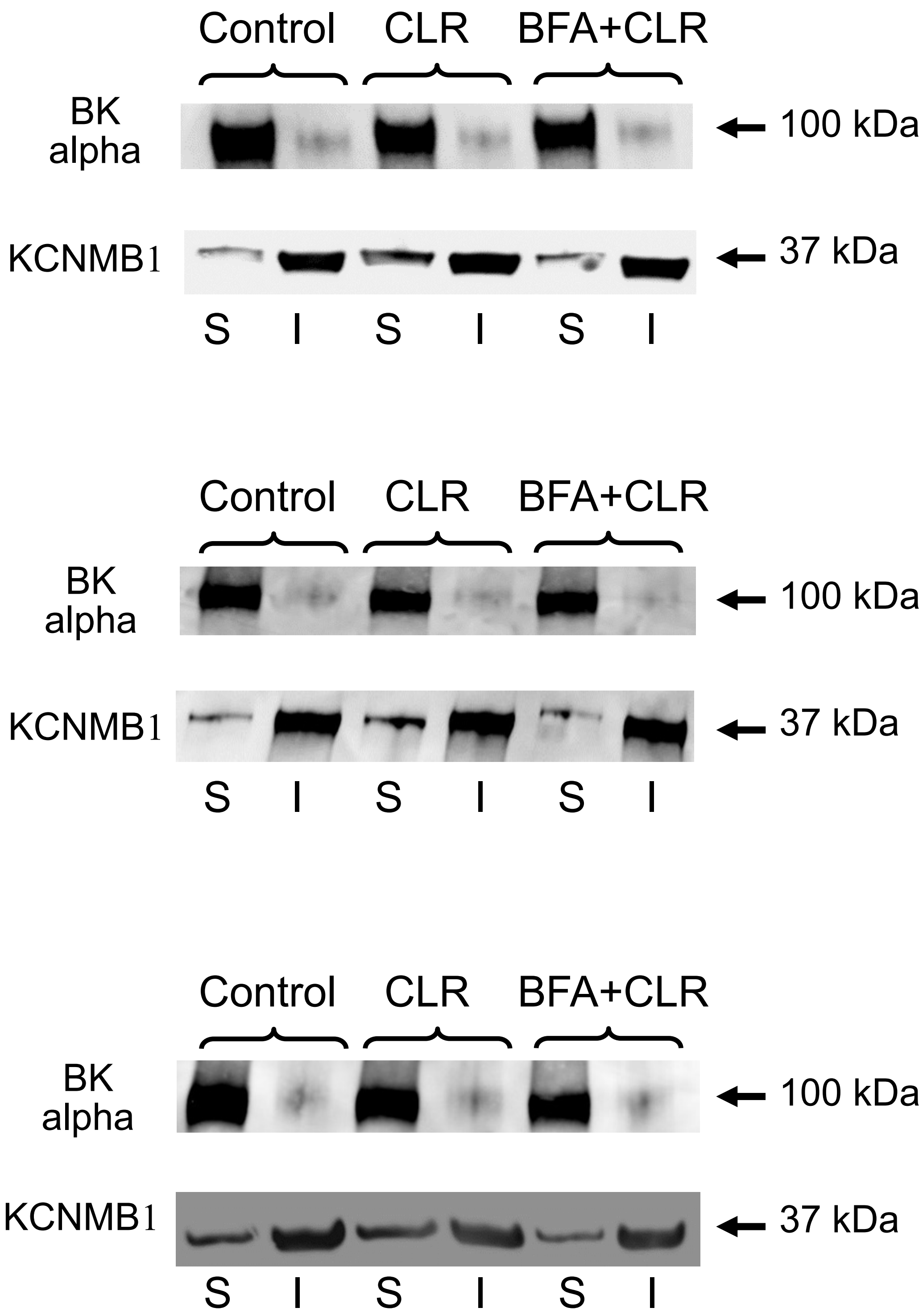

Supporting Figure 3. Western blot images obtained on independent experimental occasions show that 20 min-long incubation of rat MCA segments in CLR-enriching solution results in up-regulation of KCNMB1 cell surface (S) but not intracellular fraction (I). The CLR-driven up-regulation of surface KCNMB1 is blunted by 10 μM brefeldin A (BFA). Distribution of BK channel alpha subunits between S and I fractions remains unchanged by either treatment.
